# Positive central-mixed venous oxygen saturation gradients: high oxygen saturation in the inferior vena cava confirms high splanchnic oxygen extraction

**DOI:** 10.1186/cc9462

**Published:** 2011-03-11

**Authors:** A Reintam Blaser, T Correa, S Djafarzadeh, M Vuda, J Takala, MW Dünser, SM Jakob

**Affiliations:** 1 University of Tartu, Estonia; 2 Inselspital, University of Bern, Switzerland

## Introduction

Central venous oxygen saturation (ScvO_2_) is increasingly used as a surrogate for mixed venous oxygen saturation (SvO_2_). On average, there is a positive gradient between ScvO_2 _and SvO_2 _that has been explained by the low inferior vena cava saturation (SivcO_2_). We aimed to clarify the dynamics and associations between different venous saturations in an experimental setting of porcine peritonitis.

## Methods

Thirty-two anaesthetized pigs (40.3 ± 3.8 kg (mean ± SD)) were randomly assigned (*n *= 8 per group) to a nonseptic control group or one of three septic groups in which the pigs were observed for 6, 12 or 24 hours. Thereafter, resuscitation was performed for 48 hours. The pulmonary artery, superior vena cava and inferior vena cava (IVC) were catheterized. The catheter for IVC measurements was placed 5 cm below the diaphragm. SvO_2_, ScvO_2 _and SivcO_2 _were measured at 12-hour intervals starting at study baseline. Differences between saturations at different time points were tested with a *t *test for paired measurements.

## Results

**Figure 1:**
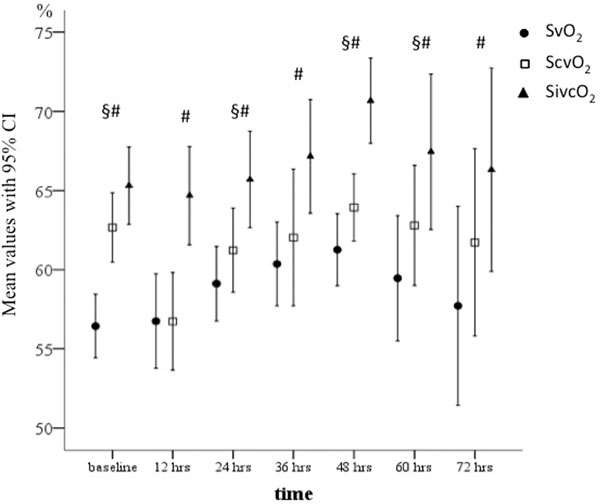
**Dynamics of mixed venous, superior and inferior vena cava saturations**. §Difference between SvO_2 _and ScvO_2_, *P *< 0.05. *Difference between SvO_2 _and SivcO_2_, *P *< 0.005.

One hundred and ninety-two (136 in septic and 56 in control animals) simultaneous measurements of SvO_2_, ScvO_2 _and SivcO_2 _were analysed. Mean SvO_2 _was 58.7 ± 7.2%, ScvO_2 _61.5 ± 8.3% and SivcO_2 _66.7 ± 8.5%. Dynamics of the saturations throughout the study are presented in Figure 1. ScvO_2 _was numerically higher than SvO_2 _in 133 (69.3%) of all measurements. In 122 of these 133 measurements (91.7%), SivcO_2 _exceeded SvO_2 _as well.

## Conclusions

In most of the measurements, both ScvO_2 _and SivcO_2 _were higher than SvO_2_. Our results suggest a high oxygen extraction of splanchnic organs as the reason for positive ScvO_2_-SvO_2 _gradients.

